# Novel Visualization Methods Assisted Transurethral Resection for Bladder Cancer: An Updated Survival-Based Systematic Review and Meta-Analysis

**DOI:** 10.3389/fonc.2021.644341

**Published:** 2021-07-13

**Authors:** Honglin Li, Yubin Cao, Pingchuan Ma, Zhongkai Ma, Chunjie Li, Wenbin Yang, Lingyun Zhou

**Affiliations:** ^1^ State Key Laboratory of Oral Diseases, National Clinical Research Center for Oral Diseases, West China School of Stomatology, Sichuan University, Chengdu, China; ^2^ Department of Head and Neck Oncology, West China Hospital of Stomatology, Sichuan University, Chengdu, China; ^3^ Department of Oral and Maxillofacial Surgery, West China Hospital of Stomatology, Sichuan University, Chengdu, China; ^4^ Department of Medical Affairs, West China Hospital of Stomatology, Sichuan University, Chengdu, China; ^5^ Center of Infectious Diseases, West China Hospital of Sichuan University, Chengdu, China

**Keywords:** narrow-band imaging, photodynamic diagnosis, hexylaminolaevulinate, 5-aminolaevulinic acid, cystectomy, cystoscopy

## Abstract

**Background:**

Photodynamic diagnosis and narrow-band imaging could help improve the detection rate in transurethral resection (TUR) of bladder cancer. It remained controversial that the novel visualization method assisted transurethral resection (VA-TUR) could elongate patients’ survival compared to traditional TUR.

**Methods:**

We performed electronic and manual searching until December 2020 to identify randomized controlled trials comparing VA-TUR with traditional TUR, which reported patients’ survival data. Two reviewers independently selected eligible studies, extracted data, assessed the risk of bias. Meta-analysis was conducted according to subgroups of types of visualization methods (A) and clinical stage of participants. Publication bias was detected.

**Results:**

We included 20 studies (reported in 28 articles) in this review. A total of 6,062 participants were randomized, and 5,217 participants were included in the analysis. Only two studies were assessed at low risk of bias. VA-TURB could significantly improve the recurrence-free survival (RFS) (HR = 0.72, 95% CI: 0.66 to 0.79, P <0.00001, I^2^ = 42%) and progression-free survival (PFS) (HR = 0.62, 95% CI: 0.46 to 0.82, P <0.0008, I^2^ = 0%) compared with TUR under white light. The results remain stable whatever the type of visualization method. The difference could be observed in the non-muscle-invasive bladder cancer (NMIBC) population (P <0.05) but not in the mixed population with muscle-invasive bladder cancer (MIBC) participants (P >0.05).

**Conclusion:**

VA-TUR could improve RFS and PFS in NMIBC patients. No significant difference is found among different types of VA-TUR. VA-TUR may be not indicated to MIBC patients.

## Introduction

Bladder cancer is the fourth most common cancer in men (1). Nine men in every 100,000, and 2.2 as many women worldwide develop bladder cancer (2). According to the invasion of the muscle layer, bladder cancer can be divided into non-muscle invasive bladder cancer (NMIBC) and (MIBC). Of newly diagnosed tumors, 70% are NMIBC comprised of stages Ta, T1 and carcinoma *in situ* (CIS) (3). Transurethral resection (TUR) after white light cystoscopy is taken as the primary treatment option for NMIBC (4). Of particular concern is the fact that despite resection assisted by the current gold standard-white light cystoscopy, patients still have a high probability of recurrence more than 50% (5, 6). It is widely shared that a complete resection plays a central role in achieving a good prognosis (7). Residual tumors are found in 33–76% of patients and are usually located at the border of the resected tumor (8, 9). Although the bladder is a perfect darkroom for white light in the TUR, the detection of tumor is still limited to the visual localization and diagnosis experience of the surgeon, particularly of the small, occult tumor lesions and CIS (10). Therefore, some new technologies are being developed to visualize the missing lesions and guide tumor resection during TUR.

There are two main types of new technologies of tumor visualization currently used in clinical practice: photodynamic diagnosis (11) (PDD) and narrow-band imaging (NBI) (12). PDD in the bladder requires intravesical instillation of an exogenous photosensitizer, 5-aminolaevulinic acid (5-ALA) (13) or hexyl-aminolaevulinate (HLA) (14), which accumulate selectively in tumor cells (15). Under the light with a given wavelength, lesions emit red fluorescence among the green areas of healthy tissue. The visual contrast between tumor and healthy tissue allows surgeons to detect the lesion sensitively which appears to be normal in the white light cystoscopy, thereby achieving complete removal of the tumor. 5-ALA currently licensed by the FDA for the photodynamic treatment increases the production of the photosensitizer, PpIX (16). It is now well established from a variety of studies, that 5-ALA-induced PpIX preferentially accumulates in many cancer cells including the bladder (16). HLA is the derivative of 5-ALA to improve its bioavailability and solubility, which means NMIBC appears brighter and lasts longer in the HLA-guided TUR (17). Narrow-band imaging (NBI) is another novel endoscopic technique applied in clinical practice. NBI enhance the contrast between the superficial mucosa and microvascular structures *via* only emitting two bandwidths of illumination, 415 nm (blue) and 540 nm (green) (18, 19). Assisted by specialized cystoscopes, surgeons could notice abnormal microvasculature and perform NBI-guided TURBT without prior instillation of dyes (20).

A previous systematic review and meta-analysis suggested that novel visualization methods assisted TUR (VA-TUR) by PDD or NBI can improve cancer detection and decrease the risk of BC recurrence in the short and long term, compared with white light (20, 21). VA-TUR have been recommended as weak evidence in the latest European Association of Urology Guidelines on NMIBC (22). However, controversies remained on whether VA-TUR could elongate patients’ survival, whereas no previous studies have addressed the complete survival data of patients, to the best of our knowledge. Therefore, this systematic review and meta-analysis aimed to analyze the effects of VA-TUR on the survival of bladder cancer patients, based on the time-to-event survival data.

## Methods

This systematic review adhered to preferred reporting items for systematic review and meta-analysis (PRISMA) statement (23).

### Eligibility Criteria

Types of studies: randomized controlled trials (RCTs).

Types of participants: Patients (>18 yr) with suspected bladder cancer (first diagnosis or recurrence) based on at least one documented cystoscopy.

Types of interventions: any types of VA-TUR;

Types of controls: traditional TUR under white light or other types of VA-TUR;

Types of outcome measures: recurrence-free survival (RFS), progression-free survival (PFS), overall survival (OS), cancer-specific survival (CSS).

### Literature Search

We searched PubMed, Embase, Cochrane Central Register of Controlled Trials, and China National Knowledge Infrastructure from inception to December 2020. The search strategy of each database was provided in the supplemental materials. The search attempted to identify all relevant studies, irrespective of language. We translated non-English papers. Two researchers (HL and YC) independently screened the titles and abstracts and selected articles for full-text review. They then independently reviewed full-text articles for eligibility. A third researcher (WY) arbitrated any differences that could not be resolved by consensus. We also performed a manual search of the references of eligible articles and relevant systematic reviews.

### Data Extraction

Two review authors (HL and YC) carried out the data extraction in duplicate. Disagreements were resolved by discussion, and an arbitrator (WY) was involved when the disagreement remained unresolved. We designed a customized data extraction form, in accordance with guidance from the Cochrane Handbook for Systematic Reviews of Interventions version (24). We pilot-tested this, using a sample of the studies focusing on this topic, and then applied it to all the included studies. We collected the following data: first author, publication time, country, participants’ gender, age, study design, interventions, number of participants randomized, number of participants analyzed, clinical stage, histopathological grade, follow-up duration, recurrence state, number of tumors, size of tumors, survival data. The time-to-event survival data were extracted according to Tierney et al. if they were not reported in the full texts (25). We contacted authors for additional information regarding the survival data of individual patients.

### Assessment of Methodological Quality of Included Studies

We judged the risk of bias in each of the included studies for six domains (as identified in the Cochrane ‘Risk of Bias’ tool): random sequence generation, allocation concealment, blinding of outcome assessment, incomplete outcome data, selective reporting, and other bias (24). As blinding of participants and personnel were impossible for all studies, we did not assess this domain. For each of the following domains, we assigned a judgment of low, high, or unclear risk of bias. Two review authors (HL and YC) independently assessed the risk of bias according to the published article and the trial authors’ responses. They discussed any discrepancies, and a third review author (LZ) arbitrated when necessary.

### Method of Data Analysis

For time-to-event survival data, we calculated hazard ratio (HR) and 95% confidence intervals (CIs). We conducted Intention-to-treat (ITT) analysis if there were sufficient data. If ITT could not be adopted, we planned to analyze the available data and interpret results with caution. We used the Chi^2^ test for heterogeneity to examine if heterogeneity existed among the included studies. We used the I^2^ statistic to estimate the impact of the heterogeneity.

We used Review Manager 5 (version 5.4.1) to conduct a random-effect model inverse-variance-method meta-analysis (24). Subgroup analysis was performed according to types of visualization methods or clinical stages of the participant population. Sensitivity was further performed to examine the origin of heterogeneity. We assessed publication bias and other reporting biases with the help of funnel plots and Begg’s test if there were more than 10 trials providing results for one outcome.

### Assessment of Quality of Evidence

We used the GRADE (Grading of Recommendations, Assessment, Development and Evaluation) working group classification to assess the quality of evidence for each outcome included in the umbrella review (26). The GRADE approach categorizes evidence from systematic reviews and meta-analyses into “high,” “moderate,” “low,” or “very low” quality. Study design dictates the baseline quality of the evidence but other factors can decrease or increase the quality level.

## Results

### Results of Search

**Figure 1:**
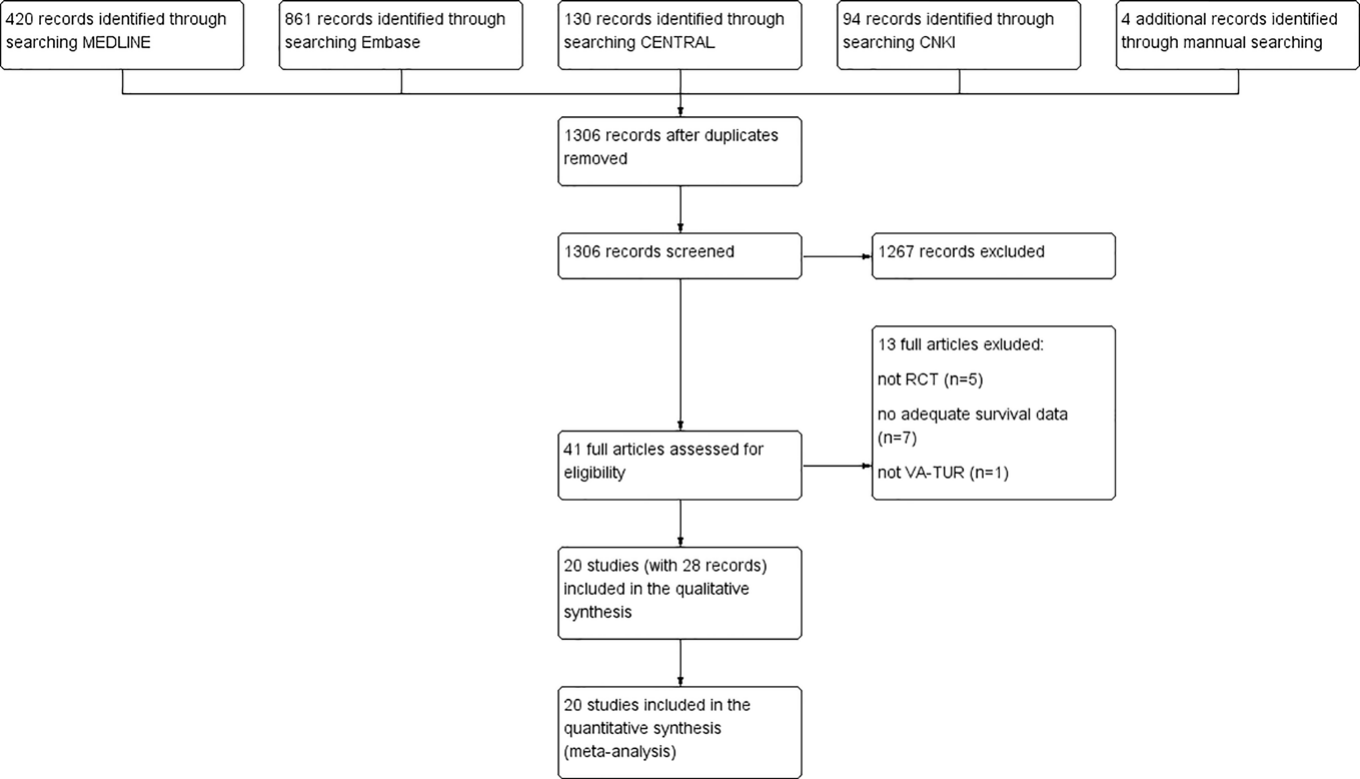
Study flow diagram.

We identified a total of 1,509 records from electronic searches and hand-searching. After removing duplicates, we screened 1,306 records by scanning the titles and abstracts. We considered 41 records to be potentially eligible and obtained the full texts for further consideration. We excluded 13 published studies. We included 20 studies (reported in 28 articles) in this review (19, 26–52). A flow diagram illustrating the study selection process is shown in **Figure 1**.

### Characteristics of Included Studies

All the included studies were two-arm parallel RCTs (**Table 1**). Six studies compared 5-ALA to WL, nine studies compared HAL to WL, and nine studies compared NBI to WL. No study directly compared different visualization methods directly. A total of 6,062 participants were randomized, and 5,217 participants were included in the analysis. The participants’ age ranged from 19 to 97 years old, and female: male = 1:2.6. Approximately 12 studies only included NMIBC patients (Stage Tis/Ta/T1), four studies also included T0 patients who were confirmed benign lesions by histopathology, and four studies included patients with muscle-invasive lesions (Stage ≥T2). Nine studies adopted the histopathological system of Grade 1/Grade 2/Grade 3, seven studies adopted the PUNLMP/Low Grade/High Grade system, and the others did not report the grade distribution. Patients in 15 studies received co-interventions of BCG/MMC/epirubicin/other adjuvant treatments. Ten studies followed participants up with one year, six studies with two to five years, four studies with five years or longer.

**Table 1:** The characteristics of included studies.

Study	Country	Design	Age (mean, range) (Y)	Gender(F/M)	No. of participants randomized	No. of participants analyzed	ITT	pT Stage	Grade	Co-intervention	Follow-up (M)	Recurrence	No. of tumors	Size of tumors
27	Czech	RCT	68.9	40/82	WL: 62 5-ALA: 60	WL: 62 5-ALA: 60	yes	Ta: 74 T1: 47	G1:63 G2:46 G3:13	/	24	P: 48 R: 74	/	1 cm: 31 1–3 cm: 67 >3 cm: 24
28	Germany; Austria	RCT	68.5 (19–89)	29/73	WL:51 5-ALA: 51	WL: 51 5-ALA: 51	yes	Ta: 80 T1: 22	G1:16 G2:74 G3:12	/	60	/	S: 50 M: 52	/
29	Romania	RCT	60.4	34/10	WL: 22 HLA: 22	WL: 22 HLA: 22	yes	Ta: 10 T1: 24	G1:13 G2:26 G3:5	MMC	12	/	/	1.84 ± 0.57 cm
30	Romania	RCT	59.8	25/88	WL: 56 HLA: 57	WL: 56 HLA: 57	yes	Ta: 33 T1: 80 Tis: 9	G1:41 G2:59 G3:13	BCG	60	/	S: 57 M: 56	/
31	Denmark	RCT	70.1	200/499	WL: 348 HLA: 352	WL: 346 HLA: 349	no	Ta: 666 T1: 23	/	BCG MMC	12	/	/	/
32	Germany	RCT	68.9 (31–89)	/	WL: 150 5-ALA: 151	WL: 103 5-ALA: 88	yes	Ta: 138 T1: 42 Tis: 11	G1:78 G2:74 G3:13	BCG MMC	90	/	S: 134 M: 57	/
36	Romania	RCT	64.1 (30–84)	59/161	WL: 110 NBI: 110	WL: 110 NBI: 110	no	Ta: 55 T1: 137	/	MMC; BCG	12	/	S: 73 M: 147	>3 cm: all
								T2: 26 T3: 2						
37	Greece	RCT	67.07	17/87	WL: 64 HLA: 66	WL: 50 HLA: 54	no	Ta: 47 T1/Tis:17	LG:51 HG:13	Epirubicin	40	P: 46 R: 58	/	/
										re-TUR				
38	Denmark	RCT	70 (35–96)	36/109	WL: 118 HLA: 115	WL: 117 HLA: 102	no	Ta: 139 T1: 5	LG:126 HG:18	/	12	/	S: 92 M: 53	<3 cm: 111 ≥3 cm: 34
39	USA	RCT	67.5 (36–99)	71/183	WL: 127 NBI: 127	WL: 127 NBI: 127	yes	T0: 95 T1: 42 Tis: 71 Ta: 47	/	BCG	24	/	S: 34 M: 220	<2 cm: 19 2–5 cm: 194 >5 cm: 41
40	Greece	RCT	64.99 (37–88)	13/73	WL: 53 HLA: 49	WL: 46 HLA: 42	no	/	LG:53 HG:23	BCG	25	P: 63 R: 23	S: 44 M: 42	/
										epirubicin				
19	Korea	RCT	/	36/118	WL: 97 NBI: 101	WL: 55 NBI: 72	no	T0: 24 Ta: 5 T1: 2 Tis: 6	LG:57 HG:64	/	12	P: 118 R: 34	/	<1 cm: 82 1–3 cm: 54 >3 cm: 16
41	Japan; Spain; Czech; UK; China; Canada; Netherlands	RCT	66.9 (18–94)	192/773	WL: 492 NBI: 489	WL: 481 NBI: 484	no	Tx: 51	G1:281 G2:248 G3:200	MMC	48	/	S: 397 M: 346	<1 cm: 545 ≥2 m: 399
								T0: 78		BCG				
								T1: 432 Tis: 19 Ta: 432						
								T2: 77						
42	Italy;	RCT	/	119/29	WL: 93 NBI: 95	WL: 72 NBI: 76	yes	Ta: 110 T1: 38	LG:80 HG:68	MMC; BCG	12	P: 83 R: 65	S: 76 M: 72	<3 cm: 108 ≥3 cm: 40
44	UK	RCT	68 (21–95)	46/183	WL: 120 HLA: 129	WL: 88 HLA: 97	no	Ta: 127 T1: 55 Tis: 1	G1:99 G2:28 G3:57	MMC BCG	12	/	S: 91 M: 149	/
46	Germany	RCT	66 (18–87)	87/290	WL: 273 5-ALA: 252	WL: 203 5-ALA: 174	no	T0: 40 Ta: 145 T1: 188 Tis: 4	G1:201 G2:107 G3:17	BCG	92	P: 255 R: 122	0–1: 171 2–7: 161 ≥8: 45	<3 cm: 289 ≥3 cm: 87
										reTUR				
47	Sweden	RCT	69.5	72/207	WL: 138 HLA: 141	WL: 138 HLA: 141	yes	T0: 33	/	MMC BCG	12	P: 136 R: 143	S: 111 M: 127	/
								Ta: 174						
								T1: 13						
								T2: 6						
								Tis: 43						
								Tx: 10						
48	Germany; Canada; USA; Netherlands	RCT	68.8	116/435	WL: 384 HLA: 382	WL: 280 HLA: 271	yes	Ta: 262 T1: 63 Tis: 41	G1+G2:422 G3: 156	BCG	9	P: 101 R: 170	/	/
51	Germany; Austria	RCT	66	100/259	WL: 189 5-ALA: 192	WL: 183 5-ALA: 187	no	Ta: 183 T1: 76 Tis: 47 T2: 18	G1:115 G2:104 G3:48	BCG	12	/	S: 216	/
													M: 143	
52	USA	RCT	76 (53–97)	33/115	WL: 300 NBI: 300	WL: 70 NBI: 78	no	Ta: 129 T1: 5 Tis: 13 T2: 1	LG:113 HG:35	/	48	P: R: 148	/	/

RCT, randomized controlled trial; WT, white light; 5-ALA, 5-aminolaevulinic acid; HAL, hexylaminolaevulinate; NBI, narrow band imaging; ITT, intention-to-treat analysis; LG, low grade; Hg, high grade; re-TUR, re-transurethral resection; BCG, Bacillus Calmette Guérin; MMC, Mitomycin-C; P, primary; R, recurrent; S, single tumors; M, multifocal tumors.

### Risk of Bias

**Figure 2:**
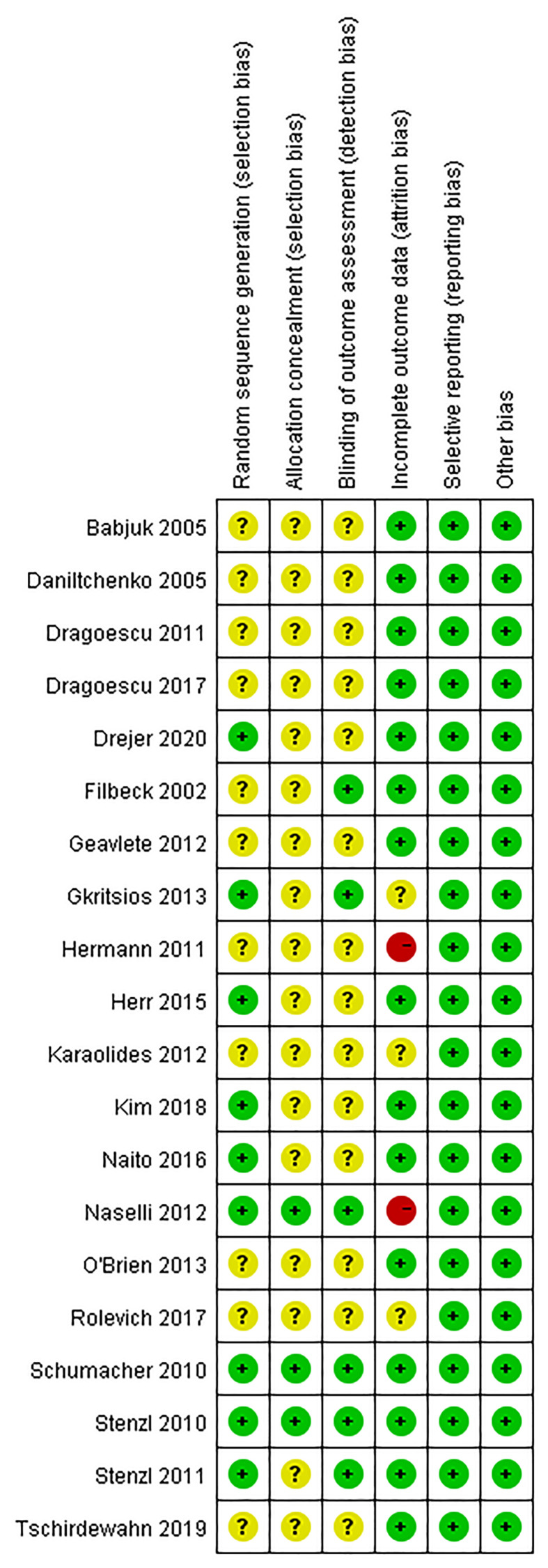
Risk of bias summary: review authors’ judgements about each risk of bias item for each included study.

Two studies were assessed at low risk of bias in all the methodological domains (**Figure 2**). Two studies were assessed at high risk of bias due to >20% of participants lost follow-up without ITT analysis. Nine studies reported the details of random sequencing methods; Three studies reported the correct allocation concealment methods; Six studies emphasized the blinding of outcome assessment; 15 studies had <10% follow-up loss or adopted ITT analysis; All the studies reported planned outcomes in suitable formats; The studies were assessed at low risk of bias in these domains. Studies were assessed at unclear risk of bias in other domains without adequate information or details. We did not identify risk of bias due to deviations from intended interventions, co-interventions, baseline balance or other sources.

### Effects of Interventions

#### RFS

**Figure 3:**
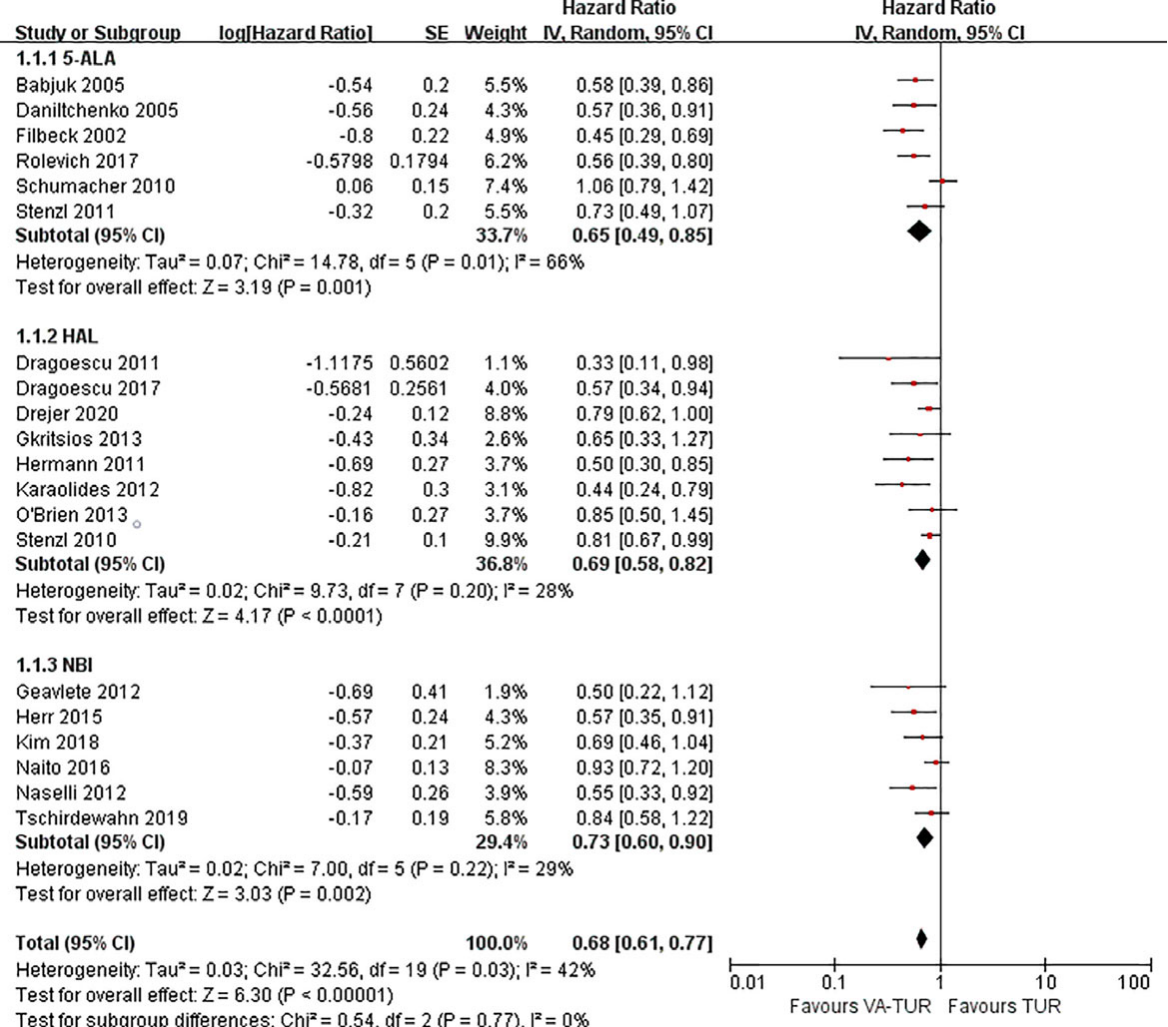
Forest plot of RFS of VA-TUR versus TUR subgrouped by types of visualization methods.

**Figure 4:**
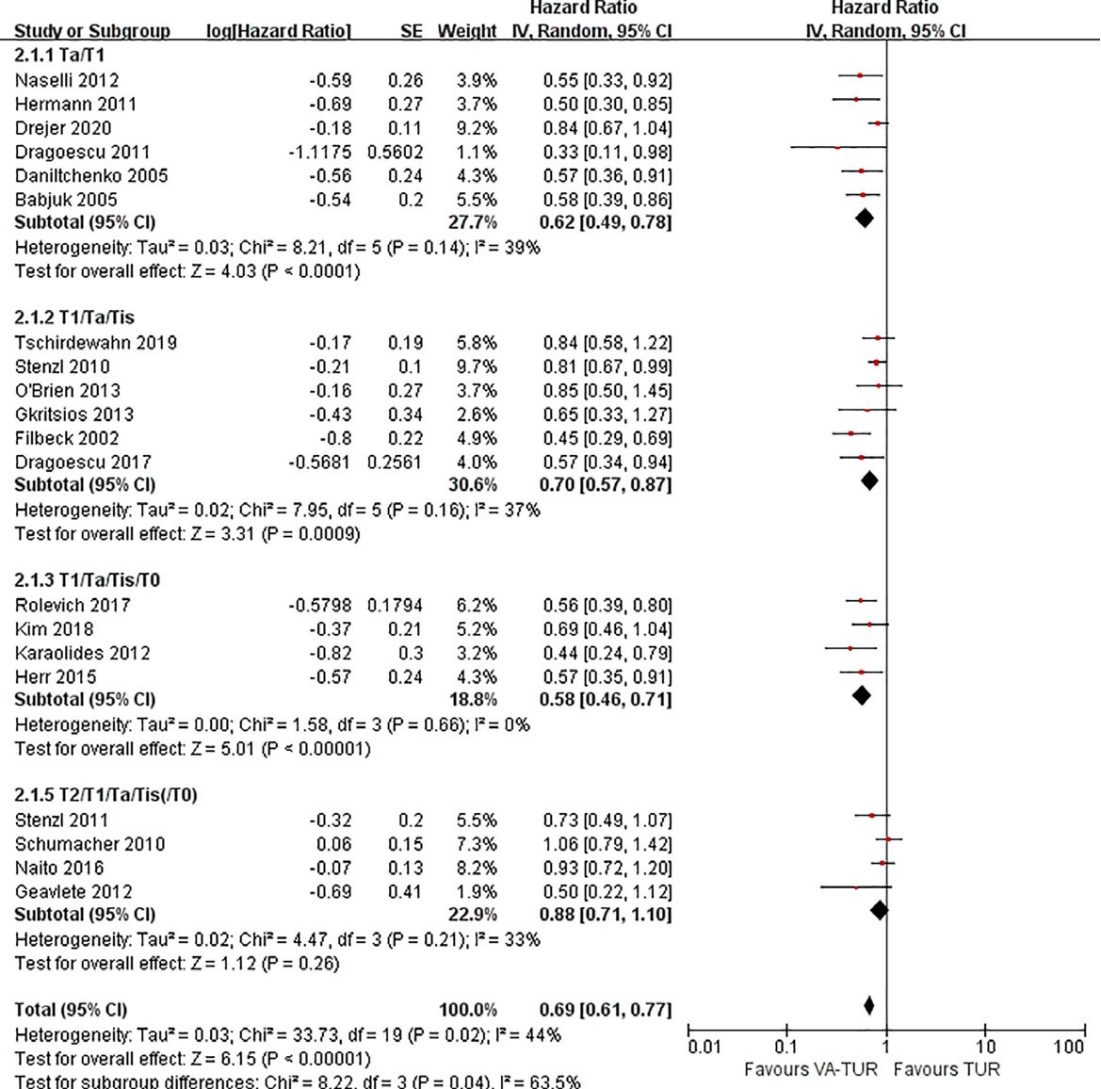
Forest plot of RFS of VA-TUR versus TUR subgrouped by clinical stage of participants.

**Figure 5:**
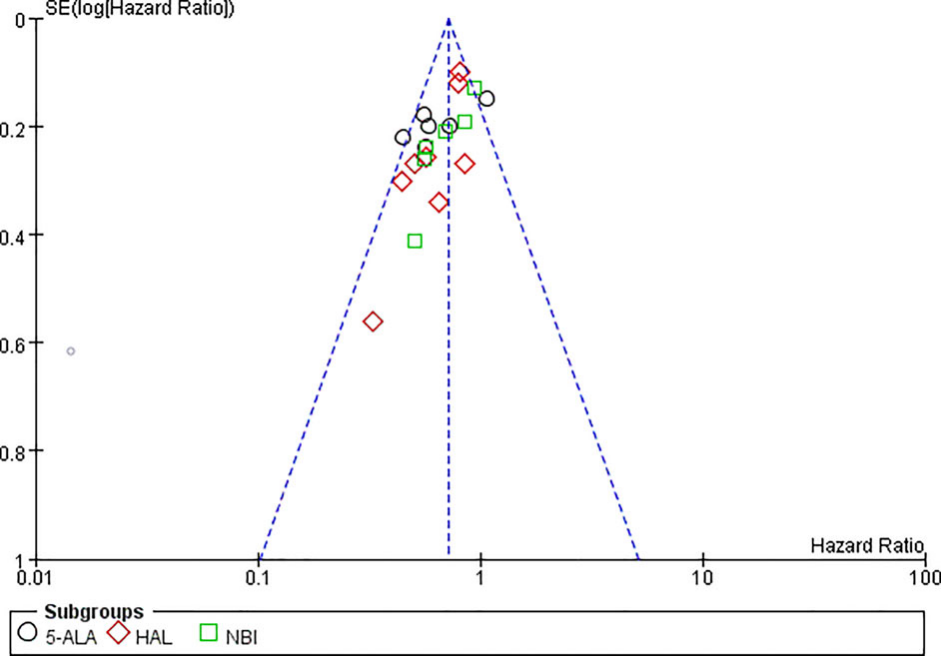
Funnel plot of RFS of VA-TUR versus TUR.

RFS could be extracted from all the included studies. VA-TURB could significantly improve the RFS compared with TUR under white light (HR = 0.72, 95% CI: 0.66 to 0.79, P <0.00001, I^2^ = 42%). Either 5-ALA, HAL or NBI could significantly improve the RFS (5-ALA: HR = 0.68, 95% CI: 0.58 to 0.79, P <0.00001, I^2^ = 66%; HAL: HR = 0.73, 95% CI: 0.64 to 0.83, P <0.00001, I^2^ = 28%; NBI: HR = 0.76, 95% CI: 0.65 to 0.90, P ≤0.001, I^2^ = 29%) and no significant subgroup differences were observed (P = 0.59, I^2^ = 0%) (**Figure 3**). RFS could be significantly improved by VA-TURB in the NMIBC population staged Ta/T1 (HR = 0.62, 95% CI: 0.49 to 0.78, P <0.0001, I^2^ = 39%), Ta/T1/Tis (HR = 0.70, 95% CI: 0.57 to 0.87, P = 0.0009, I^2^ = 37%), or T1/Ta/Tis/T0 (HR = 0.58, 95% CI: 0.46 to 0.71, P <0.00001, I^2^ = 0%), but not in the mixed population of NMIBC and MIBC (HR = 0.88, 95% CI: 0.71 to 1.10, P = 0.26, I^2^ = 33%). The subgroup differences were significant (P = 0.04, I^2^ = 63.5%) (**Figure 4**). Sensitivity analysis excluding the mixed population of NMIBC and MIBC showed no significant subgroup differences (P = 0.42, I^2^ = 0%). No publication bias was identified by funnel plot (**Figure 5**) or Begg’s test (P = 0.52).

#### PFS

**Figure 6:**
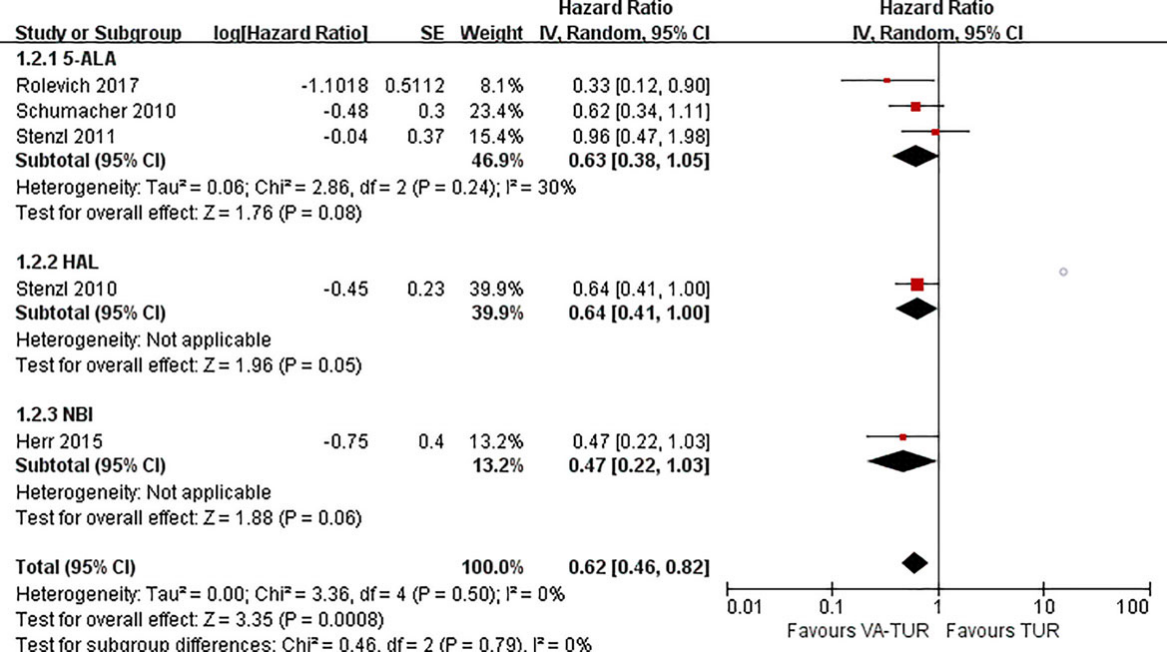
Forest plot of PFS of VA-TUR versus TUR subgrouped by types of visualization methods.

**Figure 7:**
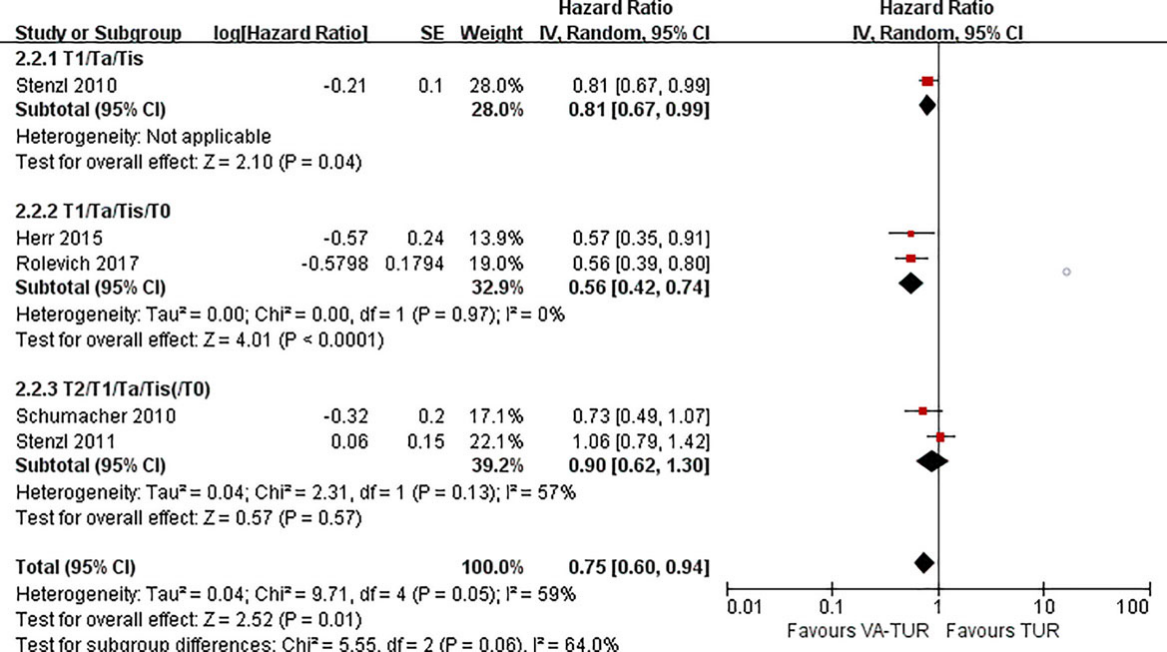
Forest plot of PFS of VA-TUR versus TUR subgrouped by types of clinical stage of participants.

PFS could be extracted from five studies, three of 5-ALA, one of HAL, and one of NBI. VA-TURB could significantly improve the PFS compared with TUR under white light (HR = 0.62, 95% CI: 0.46 to 0.82, P <0.0008, I^2^ = 0%). The subgroup analysis could not show the significant effects of either 5-ALA, HAL or NBI (P >0.05) (**Figure 6**). PFS could be significantly improved by VA-TURB in the NMIBC population staged Ta/T1/Tis (HR = 0.81, 95% CI: 0.67 to 0.99, P = 0.04, I^2^ = 39%) or T1/Ta/Tis/T0 (HR = 0.56, 95% CI: 0.42 to 0.74, P <0.0001, I^2^ = 0%), but not in the mixed population of NMIBC and MIBC (HR = 0.90, 95% CI: 0.62 to 1.30, P = 0.57, I^2^ = 57%) (**Figure 7**). Sensitivity analysis and publication bias test were not performed due to limited included studies.

#### OS and CSS

Only one study reports OS and CSS. No significant benefit of OS and CSS was observed in 5-ALA assisted TUR compared with traditional white light (OS: HR = 0.69, 95% CI: 0.45 to 1.05, P = 0.09; CSS: HR = 0.55, 95% CI: 0.19 to 1.63, P = 0.28).

### Quality of Evidence

Evidence of VA-TURB improving RFS was downgraded to moderate-quality evidence due to the serious risk of bias. Evidence of VA-TURB improving PFS was downgraded to low-quality of evidence due to serious risk of bias and inconsistency. Evidence for OS and CSS was downgraded to very low-quality due to serious risk of bias, very serious imprecision and a single trial.

## Discussion

### Summary of Findings

Recent guidelines recommended that surgeons use methods to improve tumor visualization (fluorescence cystoscopy, narrow-band imaging) during TURB, but the strength was rated as weak (22). It was possibly attributed to the deficiency of firm evidence of patients’ benefits. Only limited studies reported the hazard ratio of survival, but most studies reported the test results or survival curve. Our study is the first systematic review that analyzed survival data in the time-to-event form. There is moderate-quality evidence from 20 RCTs that visualization assisted TURB may lead to an increase in RFS compared to TURB in white light, and low-quality evidence from five RCTs suggested the benefit of VA-TURB on PFS. These results indicated that VA-TURB elongated the time to first recurrence or progression.

As we are clear that VA-TURB is recommended, the next question is which visualization method is the most recommended. We noticed that the effect size of NBI was lower than 5-ALA and HAL. However, the answer may remain unknown according to current evidence. First, no significant difference was found among subgroups of 5-ALA, HAL, and NBI. Second, we noticed that two low-risk-of-bias studies, Schumacher et al. (47) and Stenzl et al. (48), showed an insignificant or critically significant result for 5-ALA (HR = 1.06, 95% CI: 0.79 to 1.42) and HAL (HR = 0.81, 95% CI: 0.67 to 0.99) respectively. The results from low-risk-of-bias studies indicated that the effects of 5-ALA or HAL might not overwhelm NBI.

Another emerging question is whether VA-TURB did not improve survival as the high-quality studies showed. We conducted a subgroup and sensitivity analysis which might elucidate this issue. We found all the studies recruiting MIBC patients did not show a significant benefit in both RFS and PFS. For RFS, the inter-subgroup heterogeneity I^2^ drastically decreased from 63.5 to 0%. These results suggested that MIBC might not be indicated to VA-TURB.

### Implications for Practice and Research

Our results suggest that surgeons should use VA-TURB for all the NMIBC patients. The type of VA-TURB could be selected in accordance with the hospital’s finance and clinician’s preference. We recommended surgeons insist on radical cystectomy for MIBC patients rather than transurethral resection according to currently available evidence.

We identified some evidence gaps *via* this systematic review, which should be filled by future researchers. First, no low-risk-of-bias studies were performed in the NMIBC population, which should be cautiously designed and conducted by future studies for 5-ALA, HAL, and NBI respectively. If the results remain stable and promising in high-quality studies, it may be acceptable to upgrade the recommendation strength of VA-TURB to strong.

Second, there were no RCTs directly compared the efficacy of 5-ALA, HAL, and NBI. Burger et al. retrospectively reviewed the patients’ records and found no difference between 5-ALA and HAL (53). A previous network meta-analysis suggested that 5-ALA might be more effective than HAL in decreasing recurrence rate (54). However, it seemed different in our study as no inter-subgroup difference was observed. Direct comparisons are needed to determine the most effective visualization method.

Third, more coefficient factors could be further explored. A post-hoc analysis by Rolevich et al. disclosed that VA-TURB might promise more patients’ survival when less experienced surgeons conducted the TURB (45). Denzinger et al. analyzed the RFS stratified by prognostic risk groups, which showed that patients of all grades profit from VA-TURB, whereas the low-risk group might obtain more compared to traditional TURB (34). Future researches could address the potential correlated factors to further refine the indications of VA-TURB.

Fourth, the effects of VA-TURB in pT2 participants still need more exploration. Our results found that VA-TURB performed similarly to traditional transurethral resection in the mixed population of NMIBC and MIBC participants. The results might have been attributable to the invalidity of transurethral resection instead of visualization methods for pT2 participants. The National Comprehensive Cancer Network guidelines list transurethral bladder tumor resection as one of the treatment options for non-cystectomy candidates (55). Moreover, a previous systematic review concluded that treatment based on a combination of resection, chemotherapy, and radiotherapy as bladder-sparing strategies may be considered as a reasonable treatment option in properly selected patients (56). However, RCTs composed with MIBC patients were very limited, this result may need further confirmation by high-quality RCTs.

In addition, although NMIBC is often recurrent, tumor progression and patient death remained to be important prognostic events. We strongly suggest researchers analyze and report the RFS, PFS, OS, and CSS completely.

### Agreements, Disagreements, and Our Strength

Previous systematic reviews or meta-analyses addressed the recurrence rate measured at 1-year follow-up, 2-year follow-up, or the longest follow-up. They all concluded that VA-TURB could reduce the recurrence rate, which was inconsistent with our study. However, in this updated systematic review, we improved the evidence credibility in several aspects. First, previous studies analyzed the recurrence as dichotomous data, which significantly sacrificed the time-to-event survival information. We extracted the time-to-event data from the survival curve or reported P values, which made meta-analysis based on survival data feasible. Second, we noticed that the inclusion of former systematic reviews was not always covered by updated review previously. Despite the comprehensive searching strategy, we manually checked the reference lists of eligible studies and relevant systematic reviews, which avoided ignoring potentially included studies. Third, previous studies did not identify the effects of staging. As the clinical staging is a significant prognostic factor of bladder cancer, we disclosed the role of staging in the survival-based analysis.

### Limitations

There are some limitations in our study. First, there may be some deviations from the true values when the HR was not reported but estimated from other information. However, we strictly performed the estimation according to Tierney’s instruction, which decreased the risk of severe deviations (25). Second, we did not perform a network analysis. However, all the RCTs compared a type of VA-TURB to TURB under white light, which constituted a star-like network. No closed loop formed, and no inconsistency between direct and indirect evidence existed, which decreased the necessity of network analysis. Third, we did not analyze the participants of different stages in an individual level. The conclusion about MIBC would be further confirmed if individual participant data of pT2 were available. Due to the limited data accessibility, we could not achieve the analysis. However, we believe the subgroup analysis may enlighten the future studies.

## Conclusions

VA-TUR could improve RFS and PFS in NMIBC patients. No significant difference is found among different types of VA-TUR. VA-TUR may be not indicated to MIBC patients.

## Data Availability Statement

The original contributions presented in the study are included in the article/**Supplementary Material**. Further inquiries can be directed to the corresponding authors.

## Author Contributions

HL: Conception, data collection, data analysis, and final approval. YC: Conception, data collection, data analysis, data visualization, data interpretation, manuscript writing, manuscript revision, and final approval. PM: Data visualization, data interpretation, and final approval. ZM: Data visualization, data interpretation, and final approval. CL: Manuscript revision and final approval. WY: Conception, manuscript revision, and final approval. LZ: Conception, manuscript revision, and final approval. All authors contributed to the article and approved the submitted version.

## Funding

This work was funded by Natural Science Foundation of China 81802468, Sichuan Science and Technology Program (2019YFS0207) and China Postdoctoral Science Foundation (2020M670062ZX) to Dr. Lingyun Zhou, and The Research and Development Foundation of West China Hospital of Stomatology, Sichuan University (RD-02-201907)to Dr. Wenbin Yang.

## Conflict of Interest

The authors declare that the research was conducted in the absence of any commercial or financial relationships that could be construed as a potential conflict of interest.
