# Supplementary material for: Novel Visualization Methods Assisted Transurethral Resection for Bladder Cancer: An Updated Survival-Based Systematic Review and Meta-Analysis
Source: Front Oncol. 2021 Jul 13;11:644341. doi: 10.3389/fonc.2021.644341 (PMC8313822; doi:10.3389/fonc.2021.644341)
Supplement: Supplementary file 1 [file DataSheet_1.docx]

**Ovid MEDLINE (N=420)**

1. exp BLADDER NEOPLASMS/
2. (bladder* adj5 (cancer* or tumour* or tumour* or neoplas* or malignant* or carcinoma*)).mp.
3. exp fluorescence/
4. fluorescence.mp.
5. exp Aminolevulinic Acid/
6. Aminolevulinic Acid.mp.
7. hexylaminolevulinate.mp.
8. exp Narrow Band Imaging/
9. Narrow Band Imaging.mp.
10. exp Indocyanine green/
11. Indocyanine green.mp.
12. randomized controlled trial.pt.
13. controlled clinical trial.pt.
14. randomized.ab.
15. placebo.ab.
16. drug therapy.fs.
17. randomly.ab.
18. trial.ab.
19. groups.ab.
20. 1 or 2
21. or/3-11
22. or/12-19
23. or/20-22
24. exp animals/ not humans.sh.
25. 23 not 24

**Embase (N=861)**

1. exp BLADDER NEOPLASMS/
2. (bladder* adj5 (cancer* or tumour* or tumour* or neoplas* or malignant* or carcinoma*)).mp
3. exp fluorescence/
4. fluorescence.mp.
5. exp Aminolevulinic Acid/
6. Aminolevulinic Acid.mp.
7. exp aminolevulinic acid hexyl ester/
8. hexylaminolevulinate.mp.
9. exp Narrow Band Imaging/
10. Narrow Band Imaging.mp.
11. exp Indocyanine green/
12. Indocyanine green.mp.
13. Randomized controlled trial/
14. Controlled clinical study/
15. random$.ti,ab.
16. randomization/
17. intermethod comparison/
18. placebo.ti,ab.
19. (compare or compared or comparison).ti.
20. ((evaluated or evaluate or evaluating or assessed or assess) and (compare or compared or comparing or comparison)).ab.
21. (open adj label).ti,ab.
22. ((double or single or doubly or singly) adj (blind or blinded or blindly)).ti,ab.
23. double blind procedure/
24. parallel group$1.ti,ab.
25. (crossover or cross over).ti,ab.
26. ((assign$ or match or matched or allocation) adj5 (alternate or group$1 or intervention$1 or patient$1 or subject$1 or participant$1)).ti,ab.
27. (assigned or allocated).ti,ab.
28. (controlled adj7 (study or design or trial)).ti,ab.
29. (volunteer or volunteers).ti,ab.
30. human experiment/
31. trial.ti.
32. (random$ adj sampl$ adj7 ("cross section$" or questionnaire$ or survey$ or database$)).ti,ab. not (comparative study/ or controlled study/ or randomi?ed controlled.ti,ab. or randomly assigned.ti,ab.)
33. Cross-sectional study/ not (randomized controlled trial/ or controlled clinical study/ or controlled study/ or randomi?ed controlled.ti,ab. or control group$.ti,ab.)
34. (((case adj control$) and random$) not randomi?ed controlled).ti,ab.
35. (Systematic review not (trial or study)).ti.
36. (nonrandom$ not random$).ti,ab.
37. (random cluster adj3 sampl$).ti,ab.
38. (review.ab. and review.pt.) not trial.ti.
39. (Random adj field$).ti,ab.
40. (we adj searched).ab. and (review.ti. or review.pt.)
41. (update adj review).ab.
42. (databases adj4 searched).ab.
43. (rat or rats or mouse or mice or swine or porcine or murine or sheep or lambs or pigs or piglets or rabbit or rabbits or cat or cats or dog or dogs or cattle or bovine or monkey or monkeys or trout or marmoset$1).ti. and animal experiment/
44. Animal experiment/ not (human experiment/ or human/)
45. 1 or 2
46. or/3-12
47. or/13-31
48. or/32-44\
49. 45 and 46 and 47 not 48

**CENTRAL (N=130)**

1. exp BLADDER NEOPLASMS/
2. (bladder* adj5 (cancer* or tumour* or tumour* or neoplas* or malignant* or carcinoma*)).mp.
3. exp fluorescence/
4. fluorescence.mp.
5. exp Aminolevulinic Acid/
6. Aminolevulinic Acid.mp.
7. hexylaminolevulinate.mp.
8. exp Narrow Band Imaging/
9. Narrow Band Imaging.mp.
10. exp Indocyanine green/
11. Indocyanine green.mp.

**CNKI (N=94)**

Note: The search terms were in Chinese and similar to those in other databases. It could be accessed via contacting corresponding authors.
